# A Retrospective Case-Control Analysis of the Outpatient Expenditures for Western Medicine and Dental Treatment Modalities in CKD Patients in Taiwan

**DOI:** 10.1371/journal.pone.0088418

**Published:** 2014-02-12

**Authors:** Ren-Yeong Huang, Yuh-Feng Lin, Sen-Yeong Kao, Yi-Shing Shieh, Jin-Shuen Chen

**Affiliations:** 1 Department of Periodontology, School of Dentistry, Tri-Service General Hospital, National Defense Medical Center, Taipei, Taiwan; 2 Division of Nephrology, Department of Internal Medicine, Tri-Service General Hospital, National Defense Medical Center, Taipei, Taiwan; 3 Division of Nephrology, Department of Medicine, Shuang Ho Hospital, New Taipei City, Taiwan; 4 Graduate Institute of Clinical Medicine, Taipei Medical University, Taipei, Taiwan; 5 School of Public Health, National Defense Medical Center, Taipei, Taiwan; 6 Department of Oral Diagnosis, School of Dentistry, Tri-Service General Hospital, National Defense Medical Center, Taipei, Taiwan; Mario Negri Institute for Pharmacological Research and Azienda Ospedaliera Ospedali Riuniti di Bergamo, Italy

## Abstract

**Background:**

To determine if expenditures for dentistry (DENT) correlate with severity of chronic kidney disease (CKD).

**Methods:**

A total of 10,457 subjects were enrolled from January 2008 to December 2010, divided into three groups: healthy control (HC) group (n = 1,438), high risk (HR) group (n = 3,392), and CKD group (n = 5,627). Five stages were further categorized for the CKD group. OPD utilization and expenditures for western medicine (WM), DENT, and TCM (traditional Chinese medicine) were analyzed retrospectively (2000–2008) using Taiwan's National Health Insurance Research Database. Three major areas were analyzed among groups CKD, HR and HC in this study: 1) demographic data and medical history; 2) utilization (visits/person/year) and expenditures (9-year cumulative expenditure, expenditure/person/year) for OPD services in WM, DENT, and TCM; and 3) utilization and expenditures for dental OPD services, particularly in dental filling, root canal and periodontal therapy.

**Results:**

OPD utilization and expenditures of WM increased significantly for the CKD group compared with the HR and HC groups, and increased steadily along with the severity of CKD stages. However, overall DENT and TCM utilization and expenditures did not increase for the CKD group. In comparison among different CKD stages, the average expenditures and utilization for DENT including restorative filling and periodontal therapy, but not root canal therapy, showed significant decreases according to severity of CKD stage, indicating less DENT OPD utilization with progression of CKD.

**Conclusions:**

Patients with advanced CKD used DENT OPD service less frequently. However, the connection between CKD and DENT service utilization requires further study.

## Introduction

Chronic kidney disease (CKD) affects an increasing number of people around the world, and the prevalence of CKD appears to have increased over the past decade [1]. Evidence from the United States Renal Data System 2011 suggests that from the year 2000, Taiwan has had the highest incidence and prevalence of end-stage renal disease (ESRD) among all of the countries examined, with approximately 400 per million of the population affected [2], and ESRD is one of the leading causes of death in Taiwan [3]. In response, the government of Taiwan has launched a project of multidisciplinary care for CKD patients since 2004. It has been demonstrated that CKD is linked to many morbidities, creating a heavy burden on the medical insurance system [4]. Expenditures for CKD create significant economic burdens on patients as well and have become a major challenge for medical care systems [5]. Nevertheless, in light of the health-related expenditures, CKD treatment has been shown to be cost effective as it slows disease progression and prevents the development of comorbidities [5], [6].

CKD, a complex comorbid condition with multiple manifestations, is closely linked with cardiovascular disease, hypertension, anemia, diabetes, malnutrition, dyslipidemia, bone and mineral disorders, all of which increase the chances of morbidity, mortality, and healthcare costs [2], [7], [8]. In recent years, numerous studies have demonstrated higher rates of oral pathology in CKD patients with one or more oral symptoms; thus, a variety of changes occur in the oral cavity are strongly correlated with CKD itself or with CKD therapy [9], [10], [11]. In addition, poor oral health status is closely associated with markers of malnutrition, inflammation and increased risk of death for patients undergoing hemodialysis [12], [13]. Although the exact causality between diseases is intricate [11], [14], studies have demonstrated that poor oral health conditions and its severe consequences are closely associated with the incidence or progression of CKD [15], [16], [17]. Accordingly, it is widely accepted that CKD can have a critical impact on oral health; likewise, poor oral health has been linked to CKD [11].

Treatment of CKD through multidisciplinary approaches may improve patient outcomes and be cost-effective [6], [18], [19], [20]. On the basis of these findings, it should be emphasized that monitoring and maintaining the oral health status of CKD patients, as well as in patients who are considered for renal dialysis or as transplant candidates is essential. This would justify an increased attention to and better awareness of dental care in CKD patients. Furthermore, it might be possible to achieve better clinical and economic outcomes for CKD patients if patients are comprehensively evaluated and referred to the relevant specialty early, including dental services. However, to date, there is no retrospective epidemiologic study from a general population performed by analyzing a nationwide hospital-based database to investigate the relationship between the utilization and expenditures of dental services and CKD progression.

Recent publications focusing on medical care expenditures in CKD have concentrated mainly on Western Medicine (WM), including hospitalization [21], [22], pharmacy services [23], [24] and individual co-morbidity costs [4], [25], [26], [27], [28], [29]. Despite the emerging studies that have investigated possible associations between oral health and CKD [11], the correlation between Dentistry (DENT) and Traditional Chinese Medicine (TCM) outpatient (OPD) utilization and expenditures and the progression of kidney disease in the CKD population is largely unknown.

To the best of our knowledge, there are no large, hospital-based studies which outline the relationship between DENT and TCM utilization and expenditures for CKD patients. The objective of this study was designed to use a nationwide case control cohort to investigate DENT OPD utilization and associated expenditures in patients at various stages of CKD.

## Methods

### Study design and populations

**Figure 1:**
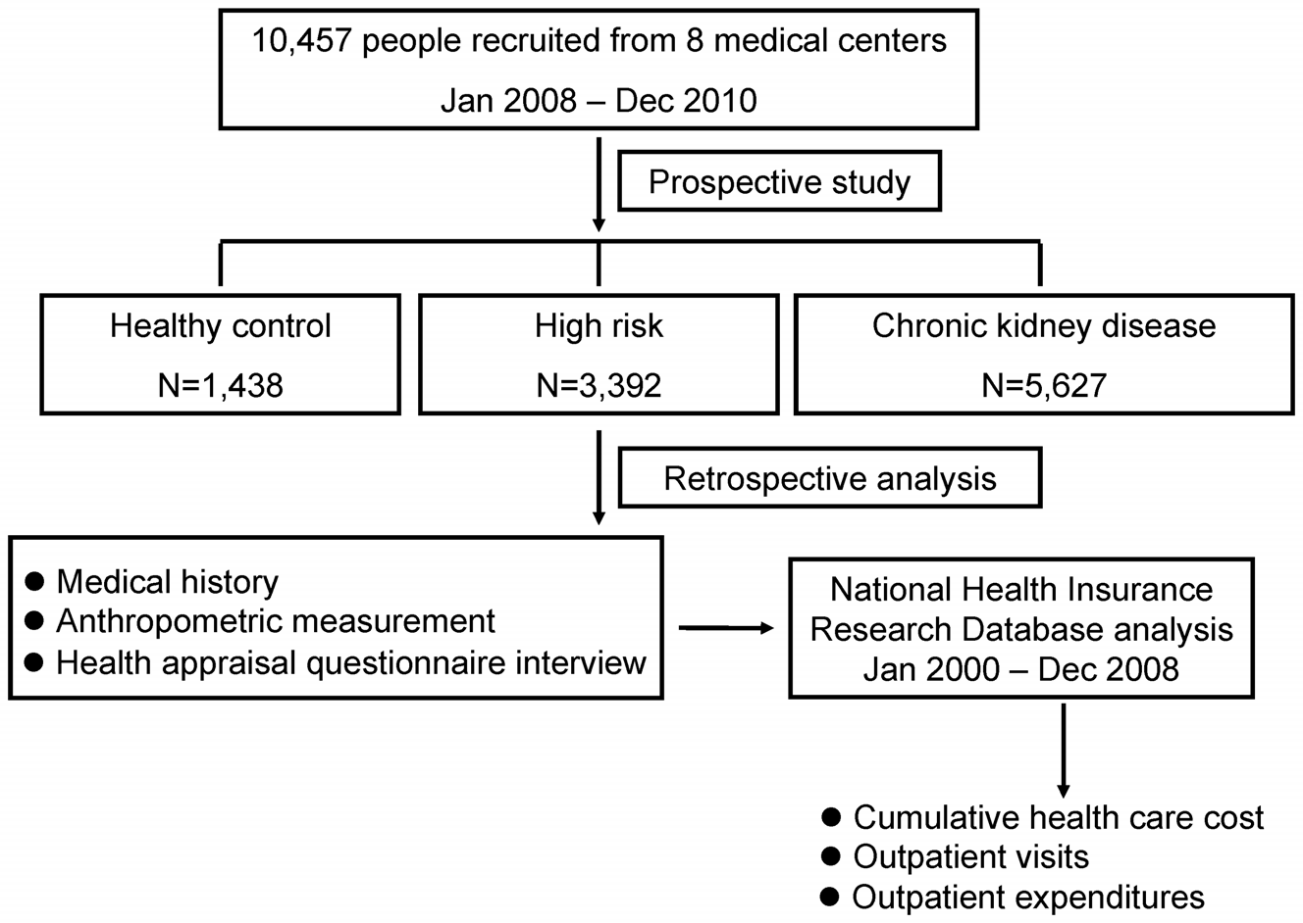
Flow chart of the selection process of the study participants.

A case-control study was conducted over 3 years. Three study groups, including healthy control (HC), high risk (HR) and chronic kidney disease (CKD), were collected throughout the period January 1, 2008, to December 31, 2010. A total of 10,457 Taiwan people all covered by the National Health Insurance Program (NHIP) from 8 medical centers located in different regions of Taiwan were the subjects of this study. Participants recruited for this study were randomly selected from the participating medical centers. The design for this study is a cluster randomized without age- or gender-match. This kind of design is vulnerable to lack of comparability; however, this design makes it easy to increase sample size and calculate expenditure more accurately. A detailed medical history, anthropometric measurements, laboratory analyses, and a health appraisal questionnaire eliciting demographic, socioeconomic and behavioral risk factors were conducted through face-to-face interviews with each participant by well-trained investigators at the initial visit. Written informed consent was obtained from all study participants. At the end of the three-year study, all participants' claims data were analyzed, and their OPD utilization and expenditure, particularly in WM, DENT, and TCM, were analyzed retrospectively from the National Health Insurance Research Database (NHIRD). A flow chart summarizing the selection process of the study participants is given in Figure 1. This study protocol involving human subjects was reviewed and approved by the Institutional Review Board of Tri-Service General Hospital, National Defense Medical Center and other participating medical centers.

### Definition of participants

All eligible participants were categorized into 3 mutually exclusive categories: “HC”, “HR”, and “CKD,” based on estimated glomerular filtration rate (eGFR) and medical history. The level of eGFR was calculated using the Modification of Diet in Renal Disease study equation [30].

Individuals in the HC group, eGFR ≥60 (mL/min/1.73 m^2^) without renal abnormalities or family history of renal diseases, were recruited from health examination in the communities or participating hospital-affiliated health evaluation units.

Patients in the HR group were eGFR ≥60 (mL/min/1.73 m^2^) and had to meet one of the following criteria: (1) diagnosed diabetes mellitus (DM), hypertension, cardiovascular disease; (2) family member diagnosed with CKD or receiving dialysis treatment.

The CKD stages were defined according to clinical practice guidelines developed under the Kidney Disease: Improving Global Outcomes (KDIGO) classification system established by the National Kidney Foundation [31], with further classification of stage 3 disease into stage3a (eGFR <60 and ≥45 mL/min/1.73 m^2^) and stage3b (eGFR <45 and ≥30 mL/min/1.73 m^2^) [32].

### Basic data collection

A face-to-face interview was conducted to obtain participants' information regarding socioeconomic status (gender, age, residence district, occupation, household income, marital status and education level) and oral health behavior (alcohol consumption, betel nut chewing and cigarette smoking habits). The geographic locations of residency were grouped into three categories of northern, central, and southern Taiwan.

Anthropometric evaluation included measurements of wrist circumference, body weight and height to calculate body mass index (BMI). According to the Bureau of Health Promotion, Department of Health, Taiwan, BMI less than 18.5 was defined as underweight, 18.5–24 as normal, between 24 and 27 as overweight, and higher than 27 as obese [33].

### Retrospective analysis of past 9 years OPD utilization and expenditure of WM, DENT and TCM

#### Data source and validation

This hospital-based study recruited individuals from the NHIRD provided by the Bureau of National Health Insurance (BNHI), and released by National Health Research Institutes (NHRI), Miaoli, Taiwan (http://www.nhri.org.tw/nhird/). Taiwan initiated the National Health Insurance (NHI) program in March 1995 to offer affordable medical care for all residents. In addition, Taiwan has the highest incidence and prevalence of end-stage renal disease globally [2]. In response, the government of Taiwan launched a project of multidisciplinary care for CKD patients in 2004. This service is available throughout Taiwan and is covered by the NHI program. Furthermore, dental care is widely available and covered by the NHI program in Taiwan.

A distinctive characteristic of the NHIRD is its comprehensive coverage of 99% of the population, for whom the NHI program has provided universal medical coverage, comprehensive benefits, and unrestricted access to any medical institution of the patient's choice [34]. Moreover, regular justifications and claims of the medical charts are performed by the BNHI of Taiwan to ensure the fidelity of the coding system in the database. The dataset after merging from Taiwan's NHIRD was transcribed for further statistical analysis. Thus, NHIRD provides a good statistical representation for analyzing epidemiological profiles of the entire population of Taiwan. Several high-quality international peer-reviewed studies have been published based on the NHIRD data, supporting its validity for medical research [35], [36], [37], [38], [39].

#### Analysis of WM, DENT, and TCM utilization and expenditure

The OPD prescription and therapeutic coding system for WM (01–15, 22, 23, 81, 82, 83, 84) DENT (40–49), and TCM (60–69) of each participant were retrieved and transcribed from the NHIRD. Utilization and expenditures for WM, DENT and TCM, including cumulative medical care expenditures, annual OPD visits, and OPD expenditure per person from January 2000 to December 2008 were further analyzed.

#### Analysis of DENT expenditure and utilization

OPD expenditures and utilization of DENT were defined according to diagnostic code and NHI therapeutic procedure codes. The coding system by the NHI in Taiwan is performed according to the International Classification of Diseases, Ninth Revision, Clinical Modification (ICD-9-CM). Diagnostic and therapeutic procedure codes were used to define expenditure and utilization of the three most common DENT procedures: restorative therapy (ICD-9-CM code: 5210–5219; therapeutic code: 89001–89012), root canal therapy (ICD-9-CM code: 5220–5229; therapeutic code: 90001–90020), and periodontal therapy (ICD-9-CM code: 5230–5239; therapeutic code: 91001–91014). These patients' first ambulatory care visits for DENT treatment between January 1, 2000, and December 31, 2008, were assigned as the index date use of medical care.

### Statistical analyses

All statistical analyses were carried out using the SAS 9.13 system (SAS system for windows, version 8.2. SAS Institute Inc. Cary. NC) and SPSS 18.0 software package (SPSS Inc., Chicago, Illinois). Mean expenditures and frequency of medical care visits where appropriate were used to describe the characteristics of the study groups. Statistical differences in categorical variables and in continuous variables between the three groups were determined using the chi-square test and one-way analysis of variance (ANOVA), respectively. Differences between each group/stage were assessed by Scheffe post hoc tests. Level of statistical significance was set at P<0.05.

## Results

### Demographic differences among subjects

Among the 10,457 eligible participants, clinical diagnosis was made and three mutually exclusive patient groups were categorized. Data were collected for 1,438 patients in the HC group, 3,392 patients in the HR group, and 5,627 patients in the CKD group. The number of subjects for each CKD stage (stage 1 to stage 5) was 917 (stage 1), 1108 (stage 2), 763 (stage 3a), 780 (stage 3b), 1036 (stage 4), and 1023 (stage 5). Of all the eligible individuals, significant differences existed in demographic characteristics and socioeconomic status among groups (all p<0.001) (Table 1). The majority of the patients in the CKD group were older, with a mean age 61.04±15.21 years compared with 57.59±14.30 yrs and 46.62±15.15 yrs in the HR and HC groups, respectively. Of the analyzed socioeconomic variables, patients in the CKD group were more likely to be unemployed (56.7%), have a household income ≤40,000 NT$ (71.8%), and lower education achievement<college level (84.3%) when compared with other groups (Table 1).

**Table 1:** Demographic characteristics and socioeconomic status of eligible subjects.

	HC (n = 1,438)		HR (n = 3,392)		CKD (n = 5,627)		
Variables	n	%	n	%	n	%	*P* a
Gender							<0.001
Male	477	33.2	1,554	45.8	3,247	57.7	
Female	961	66.8	1,838	54.2	2,380	42.3	
Age (years)							<0.001
mean ±SD	46.62±15.15	57.59±14.30	61.04±15.21	<0.001			
<45	680	47.3	616	18.2	796	14.1	
45–64	551	38.3	1,589	46.8	2,285	40.6	
65–74	138	9.6	794	23.4	1,386	24.6	
>75	69	4.8	393	11.6	1,160	20.6	
Living district (area of Taiwan)							<0.001
Northern	619	43.0	1,206	35.6	2,419	43.0	
Central	413	28.7	1,127	33.2	1,373	24.4	
Southern	406	28.3	1,059	31.2	1,835	32.6	
Marital status							<0.001
Married (%)	1,017	70.7	2,754	81.2	4,496	79.9	
Single (%)	334	23.2	326	9.6	546	9.7	
Other (%)	88	6.1	312	9.2	585	10.4	
Occupation							<0.001
None	362	25.2	1,638	48.3	3,191	56.7	
Government	104	7.2	149	4.4	242	4.3	
Agriculture	11	0.8	64	1.9	135	2.4	
Business	135	9.4	319	9.4	445	7.9	
Labor	121	8.4	282	8.3	405	7.2	
Others	705	49	940	27.7	1,210	21.5	
Household income (NT$)							<0.001
None (%)	224	15.6	1,238	36.5	2,481	44.1	
<40,000 (%)	387	26.9	987	29.1	1,559	27.7	
4–90,000 (%)	520	36.2	814	24.0	1,092	19.4	
>90,000 (%)	306	21.3	353	10.4	495	8.8	
Education level							<0.001
<Junior high (%)	267	18.6	1,442	42.5	2,864	50.9	
Senior high (%)	598	41.6	1,323	39.0	1,879	33.4	
>College (%)	572	39.8	628	18.5	883	15.7	

Unless otherwise indicated, values are number (percentage). The eligible subjects were recruited patient from 2008 to 2010. N = 10,457.

Abbreviations: CKD, chronic kidney disease; HC, healthy control; HR, high risk; NT$, new Taiwan dollars.

a Chi-square test. P<0.05 was considered statistically significant.

### Family history, anthropometric measurements and oral health habits of participants

Among the eligible individuals, significant differences existed in family history among groups, with a higher prevalence of diabetes mellitus, heart diseases, and cerebrovascular diseases (CVDs) observed in CKD and HR patients than in HC patients (all p<0.001) (Table 2).

**Table 2:** Medical history, anthropometric measurements and oral habits of eligible patients.

	HC (n = 1,438)		HR (n = 3,392)		CKD (n = 5,627)		
Variables	n	%	n	%	n	%	*P* b
**Medical history**							
Family history							
Diabetes mellitus	309	21.5	984	29.0	1,412	25.1	<0.001
Heart diseases	127	8.8	271	8.0	304	5.4	<0.001
CVDs	112	7.8	265	7.8	304	5.4	<0.001
**Physical status**							
BMI (Kg/m^2^)							<0.001
Underweight^a^	97	6.8	75	2.2	214	3.8	
Normal weight^a^	679	47.2	1,275	37.6	2,223	39.5	
Overweight^a^	292	20.3	1,041	30.7	1,626	28.9	
Obesity^a^	370	25.7	1,001	29.5	1,564	27.8	
Waist (cm)							<0.001
<70	293	20.4	268	7.9	394	7	
71–80	585	40.7	862	25.4	1,311	23.3	
81–90	293	20.4	1,262	37.2	1,930	34.3	
>91	266	18.5	1,001	29.5	1,992	35.4	
**Oral habit**							
Alcohol	121	8.4	461	13.6	838	14.9	<0.001
Betel nuts	19	1.3	122	3.6	248	4.4	<0.001
Cigarette	141	9.8	638	18.8	1,283	22.8	<0.001

The eligible subjects were recruited patient from 2008 to 2010. N = 10,457.

Abbreviations: BMI, body mass index; CKD, chronic kidney disease; CVDs, cerebrovascular diseases; HC, healthy control; HR, high risk.

a Underweight: BMI<18.5; Normal weight: BMI = 18.5–24; Overweight: BMI = 24–27; Obesity: BMI>27.

b Chi-square test. P<0.05 was considered statistically significant.

The anthropometric evaluations of body mass index (BMI) and waist circumference showed significant differences among groups (p<0.001) (Table 2). Only a minority of eligible individuals were considered obese, with a BMI>27 (28.1%) and waist circumference >91 cm (31.2%) (Table 2).

Subjects' oral health habits, including alcohol and betel nut use, and cigarette smoking, all considered to have negative effects on oral health, were summarized (Table 2). The most frequent habits among all participants were cigarette smoking (19.7%), alcohol use (13.6%), and betel nut use (3.7%). Participants in the CKD and HR groups were more likely to have these oral habits than were those in the HC group (all p<0.001) (Table 2).

### OPD utilization and expenditure in WM, DENT and TCM

**Figure 2:**
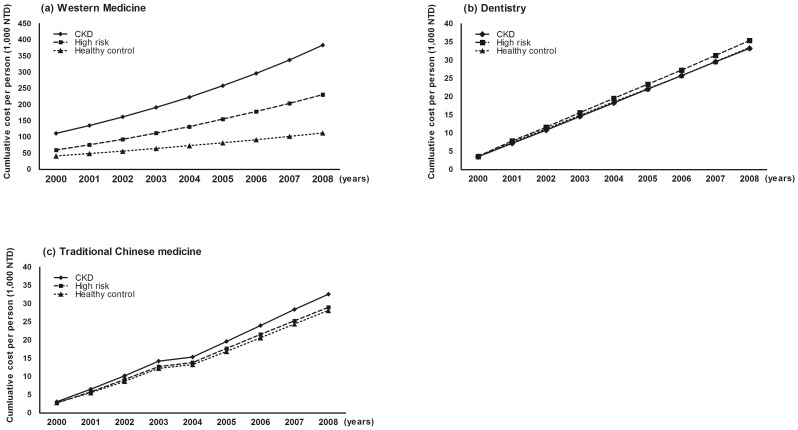
Cumulative OPD expenditures per person in WM, DENT, and TCM from 2000 to 2008. The eligible subjects were recruited from 2008 to 2010. N = 10,457. Abbreviations: CKD, chronic kidney disease; DENT, Dentistry; HC, healthy control; HR, high risk; OPD, outpatient; NT$, new Taiwan dollars; TCM, Traditional Chinese Medicine; WM, Western Medicine.

Figure 2 shows the cumulative OPD expenditures per person in WM, DENT and TCM from 2000 to 2008. In general, patients with CKD had greater overall expenditures in WM than for DENT and TCM. Interestingly, the cumulative expenditures for WM for the CKD group exhibited remarkable annual increase when compared with the HR and HC groups, whereas this tendency was not observed for DENT (Figure 2B) and TCM (Figure 2C) expenditures.

The annual number of OPD visits per person, and expenditures per person (NT$) for WM and DENT exhibited significant differences among the groups (Table 3). For WM, the CKD group had higher expenditures and OPD visits than the HC or HR groups; however, these trends were not observed for DENT (Table 3). These parameters steadily increased along with the severity of CKD stages (stage 1–5) in WM (p<0.01) (Table 3). Interestingly, only the OPD visits for DENT services showed significant differences in different CKD stages (p = 0.034) although significant differences for DENT expenditures were not found (p = 0.166) (Table 3), suggesting that DENT expenditures did not increase as the patient's kidney disease became worse.

**Table 3:** The average annual number of OPD visits and expenditures per person in WM and DENT among eligible patients and different CKD stages from 2000 to 2008.

					CKD						
Parameters	HC (n = 1,438)	HR (n = 3,392)	CKD (n = 5,627)	*p* b	Stage 1	Stage 2	Stage 3a	Stage 3b	Stage 4	Stage 5	*p* b
**Western Medicine**											
OPD visits (mean ± SD)	20.4±10.7	30.6±15.7	42.3±28.7	<0.01^c^	29.1±16.7	35.0±20.0	40.0±21.0	45.0±27.4	49.8±29.0	50.9±40.8	<0.01^e^
OPD expenditures^a^ (NT$, mean)	12,986	26,427	42,213	<0.01^c^	244,703	340,972	404,935	452,443	505,490	532,729	<0.01^e^
**Dentistry**											
OPD visits (mean ± SD)	4.1±9.2	4.9±2.7	4.7±2.0	0.019^d^	4.3±2.3	4.7±2.2	4.4±1.7	4.8±1.8	5.5±2.4	4.4±1.5	0.034
OPD expenditures^a^ (NT$, mean)	38,384	43,537	40,130	0.049	37,140	41,024	38,799	40,911	45,290	37,394	0.166

The eligible subjects were recruited patient from 2008 to 2010. N = 10,457.

Abbreviations: CKD, chronic kidney disease; DENT, Dentistry; HC, healthy control; HR, high risk NT$, new Taiwan dollars; OPD, outpatient; WM, Western Medicine.

a Expenditures were rounded to the nearest whole dollar.

b ANOVA. P<0.05 was considered statistically significant.

c Scheffe's test : CKD>HR>HC.

d Scheffe's test : HR>HC.

e Scheffe's test :Stage5>Stage4>Stage3b>Stage3a>Stage2>Stage1.

### Utilization and expenditure of DENT therapeutic procedures

Annual OPD visits and expenditures per person for restorative and periodontal therapy exhibited significant differences among groups; however, for root canal therapy, only OPD visits presented considerable difference among all groups (p = 0.0063) (Table 4). At different CKD stages, the average expenditures and OPD visits for restorative filling and periodontal therapy (all p<0.0001), but not root canal therapy, showed significant decreases according to severity of CKD stages (Table 4), indicating less DENT utilization with progression of CKD.

**Table 4:** The average annual number of OPD visits, and expenditures per person of different dental procedures among eligible patients and different CKD stages from 2000 to 2008.

					CKD						
Parameters	HC (n = 1,438)	HR (n = 3,392)	CKD (n = 5,627)	*p* b	Stage 1	Stage 2	Stage 3a	Stage 3b	Stage 4	Stage 5	*p* b
**Restorative therapy**											
OPD visits (mean ± SD)	0.81±0.66	0.83±0.72	0.78±0.69	0.0027^c^	0.83±0.74	0.82±0.69	0.8±0.67	0.81±0.7	0.77±0.74	0.64±0.59	<0.0001^h^
OPD expenditures^a^ (NT$, mean)	10,040	9,808	8,812	<0.0001^d^	9,786	9,457	9,025	8,962	8,462	7,174	<0.0001^h^
**Root canal therapy**											
OPD visits (mean ± SD)	0.38±0.34	0.43±0.42	0.42±0.39	0.0063^e^	0.43±0.36	0.43±0.42	0.41±0.35	0.45±0.41	0.43±0.42	0.41±0.34	0.436
OPD expenditures^a^ (NT$, mean)	6,179	6,522	6,140	0.0705	6,217	6,256	6,023	6,626	5,970	5,803	0.3
**Periodontal therapy**											
OPD visits (mean ± SD)	0.76±0.68	0.87±0.86	0.78±0.76	<0.0001^f^	0.76±0.71	0.84±0.78	0.83±0.73	0.86±0.76	0.76±0.84	0.67±0.66	<0.0001^i^
OPD expenditures^a^ (NT$, mean)	6,377	7,020	6,149	<0.0001^g^	6,153	6,640	6,488	6,697	5,835	5,262	<0.0001^i^

The eligible subjects were recruited patient from 2008 to 2010. N = 10,457.

Abbreviations: CKD, chronic kidney disease; DENT, Dentistry; HC, healthy control; HR, high risk NT$, new Taiwan dollars; OPD, outpatient; WM, Western Medicine.

a Expenditures were rounded to the nearest whole dollar.

b ANOVA. P<0.05 was considered statistically significant.

c Scheffe's test : HR>CKD.

d Scheffe's test : HC>HR>CKD.

e Scheffe's test : HR>CKD>HC.

f Scheffe's test : HR>CKD>HC.

g Scheffe's test : HR>HC>CKD.

h Scheffe's test : Stage1>Stage2>Stage3a>Stage3b>Stage4>Stage5.

i Scheffe's test : Stage3b>Stage2>Stage3a>Stage4>Stage5.

## Discussion

To the best of our knowledge, this is the first attempt to use a long-term, nationwide hospital-based cohort to investigate the relationship between DENT utilization and expenditures for CKD patients according to the progression of CKD stages. Our major findings were as follows: 1) group CKD demonstrated significant differences in terms of demographic data and socioeconomic performance when compared to groups HC and HR; 2) participants in group CKD had poor oral health habits compared to group HC; 3) the medical care utilization and expenditures for WM services for patients with CKD were higher when compared to groups HC and HR, but DENT and TCM services showed no significant difference among the three groups; and 4) as for DENT services, the OPD visits and expenditures of the patients receiving restorative and periodontal therapy showed a significant decrease in group CKD, but not in groups HC and HR. Moreover, the OPD visits and expenditures for group CKD decreased significantly according to the progression of CKD. All these findings provide a new understanding of the relationship between CKD and DENT services, particularly in the treatment of restorative and periodontal therapy.

First, in our study we investigated socioeconomic and demographic data, finding that group CKD was more likely to be male, unemployed or earning a low income, and more than 50% likely to have less than a junior high diploma. A US study had similar findings, in that people with CKD and limited education or low income have more risk of disability because of socioeconomic disparities [40]. Moreover, patients in our CKD group were more likely to have bad oral habits than were other groups (Table 2). A cross-sectional study regarding the oral health status of adults in Taiwan found that demographic factors (i.e., gender, marital status, and income levels) are all significantly associated with general health [41]. Thus, our findings highlight the need for more attention to DENT needs for CKD patients.

As for oral health habits, we found that group CKD had the worst habits, including alcohol use, betel nut use and smoking. It has been shown that oral health-related factors (i.e., oral hygiene, oral health status, dental care utilization, disease history, and lifestyle factors such as cigarette smoking, alcohol use, and betel nut chewing) are significantly associated with general and oral health [41]. A higher rate of concurrent usage of oral substances, particularly in the CKD group, indicates certain lifestyle patterns, which may confer a higher health risk [41]. However, previous studies demonstrated an inverse association between alcohol consumption and renal dysfunction [42], [43] because beneficial oxidative activity on endothelial function has a protective property for kidneys [42], [44]. Additionally, in Taiwan, CKD prevalence among betel-nut users is higher than in the non-users in all age groups [43]. The habit of betel-nut chewing may be associated with CKD, especially in males [43], [45]. It has been reported that relative risk for oral cancer among those who chew betel-nut in the Taiwanese population is 58.4 (95% confidence interval 7.6 to 447.6) [46]. Generally, oral health status is significantly related to socio-economic status and strongly correlated with oral health behaviors and even general health. We strongly recommend widespread public health care education targeting all three risky behaviors at the same time.

As for dental care, we found there was no difference in utilization and expenditure for dental care at different CKD stages, but the utilization of western medicine increased with the progression of CKD. Furthermore, the utilization and expenditure of periodontal therapy and restorative therapy both decreased with the progression of CKD stage (Table 4). But how can we explain this outcome? Recently, a survey was conducted from a representative database to examine self-reported dental status, dental care utilization, and dental insurance, by race/ethnicity [47], among community-dwelling older adults. The author found that Non-Hispanic White respondents reported better dental health, higher dental care utilization, and higher satisfaction with dental care compared to all other racial/ethnic groups. On the contrary, Chinese immigrants were more likely to report poor dental health, were less likely to report dental care utilization and dental insurance, and were less satisfied with their dental care compared to all other racial/ethnic groups [47]. Some factors including cost, physical disabilities, language barriers, dental fear and socio-psychological concerns may affect dental care service utilization by a specific population [41], [47], [48], [49]. It has been shown that those with CKD had a 25% lower likelihood of having a dental visit [HR = 0.75, 95% CI (0.64–0.88)] than those without CKD after adjustment for confounders [50]. In addition, the uremic patients demonstrated more dental problems than healthy controls and seem to develop their problems before they progressed to dialysis [51]. Treatments for CKD and dental care are widely available and inexpensive in Taiwan. Take CKD care, for example. According to Lin et al [52], the medical expenditures per subject per year in years 1997, 1998 and 1999 were US$ 129.7, 432.8 and 725.6 for CKD late stages, stages 3, 4 and 5. For dental care, from 1998 to 2005, the number of dentists at national level increased 30.5% from 8,020 to 10,465 and the population-dentist ratio decreased 22.0% (2,588 people per dentist in 1998 and 2,115 people per dentist in 2005). The percentage of insured population receiving dental service increased from 36.1% in 1998 to 40.8% in 2005. The dentist-to-population ratio (defined as the number of dentists per 10,000 people) was 5.0 in 2010 [53]. Thus, dental care for each participants is widely available and inexpensive in Taiwan.

It is essential to address the factors affecting the usage of dental care in CKD patients, as these may contribute to the progression of CKD stages. Greater attention to dental problems may be warranted during the progression of CKD to prevent deterioration of kidney function [51]. Furthermore, it is plausible that restorative and periodontal expenditure and utilization may provide contributory information on the deterioration of kidney function in patients with CKD [51]. Further studies to ascertain the nature of the association between oral health and CKD progression are needed.

This study has a few limitations that should be addressed. First, claims data were identified from the NHIRD under the principal payment code for DENT service and complete dental examination was not performed during face-to-face interview; however, to date, the decision criteria for subjects leading to dental treatment, including restorative or filling, endodontic and periodontal therapy is still judged by clinicians according to an imprecise coding system. Second, claims data may have minor inaccuracies even through these inaccuracies are rare. The accuracy of claims data of NHIRD is improved by a cross-checking system with full review by specialists. Thus, these inaccuracies would be unlikely to have significantly affected the results, considering the substantial sample size. Third, the study evaluated only the direct OPD expenditures, including WM, DENT and TCM expenditures. Information to determine the indirect economic burdens of CKD, such as work productivity loss and reduced quality of life, was not available. Furthermore, the current study may also suffer from detection bias. Indeed, it was not possible to capture the entire continuum of care of patients, as the NHIRD does not include information regarding the proportion of self-payment dental therapies such as denture fabrication, orthodontic treatment, dental implant placement and medical cosmetics treatment. Moreover, findings were based on a single integrated health system and may not be generalizable to larger populations because of hospital-based study design. A community- or population-based study will be needed to delineate the intricate relationship between CKD and oral health.

Since patients at advanced CKD stages in our study used DENT services less frequently, it would be very likely that individuals with more advanced CKD are older and less educated, and have lower income. Further multivariate regression analysis may have interesting findings regarding whether this association is dependent or not on some confounders such as demographics, socio-economic status and oral habits. In this study, from our collected data, we can offer some evidence regarding the factors on demographics, and socioeconomic status to support our conclusion. As for gender, our result showed a gender difference consistent with several previous studies in Taiwan [54], [55], [56]. It should be emphasized that 99% of Taiwan's population is covered by NHIP [34]. Thus, in terms of gender, there is no difference between the healthcare utilization for kidney disease [52], [57]. As for age, there is no obvious finding that individuals with more advanced CKD are older. For the later stages, such as CKD Stage 4 and Stage 5, the majority of CKD patients in our study were age 45–64 (32.8% and 39.4%, respectively), greater than for other age groups, including age >75 (28.6% for CKD Stage 4 and 24.20% for CKD Stage 5) (data not shown). Therefore, “Age” may not correlate to healthcare utilization and expenditure. As for demographic characteristics, such as residential district, a similar distribution pattern was found in our investigated groups (Table 1). The residential district may not have a significant impact on healthcare utilization for recruited patients because of the universal coverage of NHIP in Taiwan [34]. As for socioeconomic status, such as household income, more subjects in CKD Stage 2 (24%) indicate low or no income than those in Stage 5 (22.10%) or Stage 1 (19.7%) (data not shown). Lower socioeconomic status is a risk factor for CKD and progression to end-stage renal disease; however, consistent with another study [58], GFR decline was similar across income groups and patients with advanced CKD may not necessarily have lower income than those in other stages. For the “education level,” CKD patients at Stage 4 and Stage 5 have a higher likelihood of lower educational achievement (<Junior high) (Table 1). However, subjects aged 45–64 (40.9%) had less education than those 65–74 (34.0%), and those >75 (21.8%) (data not shown). Thus, in fact, we found individuals with more advanced CKD may not necessarily be less educated or have less income. Consequently, the demographic and socioeconomic factors may have only a limited influence on the analysis procedure and result of this study. Nevertheless, we should be cautions about the interpretation of the results; the interacting effects of these covariates on the correlation between CKD stages and healthcare utilization and expenditure still require further investigation.

Despite these limitations, this study has several strengths, including the important advantage of relying on real-world population-based data, a relatively substantial sample size, face-to-face questionnaire interview for each participant, and the availability of laboratory results to ascertain CKD stage.

## Conclusions

In conclusion, from the horizon of dental utilization and expenditures, this hospital-based research is the first to assess dental OPD utilization and expenditures in a population with CKD. Patients at advanced CKD stages used DENT services, including periodontal therapy and restorative filling, less frequently. However, a large and prospective study is warranted to clarify the connection between CKD stages and DENT utilization in CKD subjects.
